# Life’s Essential 8 score and its association with sperm quality parameters in reproductive-aged men: evidence from the Led-Fertyl cohort

**DOI:** 10.1093/hropen/hoaf059

**Published:** 2025-09-18

**Authors:** Estefanía Davila-Cordova, Albert Salas-Huetos, María Fernández de la Puente, Carla Ramos-Rodríguez, María Ángeles Martínez, Silvia Canudas, Antoni Palau-Galindo, Cristina Mestres-Solà, José María Manzanares-Errazu, Elena Sánchez-Resino, Michelle M Murphy, Jordi Salas-Salvadó, Nancy Babio

**Affiliations:** Universitat Rovira i Virgili, Departament de Bioquímica i Biotecnologia, Alimentació, Nutrició, Desenvolupament i Salut Mental (ANUT-DSM), Unitat de Nutrició Humana, Reus, Spain; Institut d’Investigació Sanitària Pere Virgili (IISPV), Reus, Spain; Institut d’Investigació Sanitària Pere Virgili (IISPV), Reus, Spain; Centro de Investigación Biomédica en Red de Fisiopatología de la Obesidad y Nutrición, Instituto de Salud Carlos III, Madrid, Spain; Universitat Rovira i Virgili, Departament de Ciències Mèdiques Bàsiques, Alimentació, Nutrició, Desenvolupament i Salut Mental ANUT-DSM, Unitat de Medicina Preventiva i Bioestadística, Reus, Spain; Department of Nutrition, Harvard T.H. Chan School of Public Health, Harvard University, Boston, MA, USA; Universitat Rovira i Virgili, Departament de Bioquímica i Biotecnologia, Alimentació, Nutrició, Desenvolupament i Salut Mental (ANUT-DSM), Unitat de Nutrició Humana, Reus, Spain; Institut d’Investigació Sanitària Pere Virgili (IISPV), Reus, Spain; Centro de Investigación Biomédica en Red de Fisiopatología de la Obesidad y Nutrición, Instituto de Salud Carlos III, Madrid, Spain; Institut d’Investigació Sanitària Pere Virgili (IISPV), Reus, Spain; Universitat Rovira i Virgili, Departament de Ciències Mèdiques Bàsiques, Alimentació, Nutrició, Desenvolupament i Salut Mental ANUT-DSM, Unitat de Medicina Preventiva i Bioestadística, Reus, Spain; Universitat Rovira i Virgili, Departament de Bioquímica i Biotecnologia, Alimentació, Nutrició, Desenvolupament i Salut Mental (ANUT-DSM), Unitat de Nutrició Humana, Reus, Spain; Institut d’Investigació Sanitària Pere Virgili (IISPV), Reus, Spain; Centro de Investigación Biomédica en Red de Fisiopatología de la Obesidad y Nutrición, Instituto de Salud Carlos III, Madrid, Spain; Department of Pharmacology, Therapeutics and Toxicology, Faculty of Veterinary, Universitat Autònoma de Barcelona, Catalonia, Spain; Department of Nutrition, Food Sciences and Gastronomy, School of Pharmacy and Food Sciences, Food Torribera Campus, University of Barcelona, Santa Coloma de Gramenet, Spain; Institute of Nutrition and Food Safety of the University of Barcelona, INSA-UB Maria de Maeztu Unit of Excellence, Santa Coloma de Gramenet, Spain; ABS Reus V. Centre d’Assistència Primària Marià Fortuny, Salut Sant Joan de Reus-Baix Camp, Reus, Spain; ABS Reus V. Centre d’Assistència Primària Marià Fortuny, Salut Sant Joan de Reus-Baix Camp, Reus, Spain; Endocrinology and Nutrition Department, University Hospital Sant Joan de Reus, Reus, Spain; Laboratory of Toxicology and Environmental Health, School of Medicine, Universitat Rovira i Virgili, IISPV, Reus, Spain; Universitat Rovira i Virgili, Center of Environmental, Food and Toxicological Technology—TecnATox, Departament of Basic Medical Sciences, Reus, Spain; Institut d’Investigació Sanitària Pere Virgili (IISPV), Reus, Spain; Centro de Investigación Biomédica en Red de Fisiopatología de la Obesidad y Nutrición, Instituto de Salud Carlos III, Madrid, Spain; Universitat Rovira i Virgili, Departament de Ciències Mèdiques Bàsiques, Alimentació, Nutrició, Desenvolupament i Salut Mental ANUT-DSM, Unitat de Medicina Preventiva i Bioestadística, Reus, Spain; Universitat Rovira i Virgili, Departament de Bioquímica i Biotecnologia, Alimentació, Nutrició, Desenvolupament i Salut Mental (ANUT-DSM), Unitat de Nutrició Humana, Reus, Spain; Institut d’Investigació Sanitària Pere Virgili (IISPV), Reus, Spain; Centro de Investigación Biomédica en Red de Fisiopatología de la Obesidad y Nutrición, Instituto de Salud Carlos III, Madrid, Spain; Universitat Rovira i Virgili, Departament de Bioquímica i Biotecnologia, Alimentació, Nutrició, Desenvolupament i Salut Mental (ANUT-DSM), Unitat de Nutrició Humana, Reus, Spain; Institut d’Investigació Sanitària Pere Virgili (IISPV), Reus, Spain; Centro de Investigación Biomédica en Red de Fisiopatología de la Obesidad y Nutrición, Instituto de Salud Carlos III, Madrid, Spain

**Keywords:** Life’s Essential 8, LE8, sperm quality, infertility, healthy lifestyle, male reproductive health

## Abstract

**STUDY QUESTION:**

Is Life’s Essential 8 (LE8) score associated with sperm quality parameters in healthy reproductive-aged men?

**SUMMARY ANSWER:**

Higher LE8 score adherence is positively associated with total sperm count, sperm concentration, total motility, and progressive motility.

**WHAT IS KNOWN ALREADY:**

Several lifestyle and cardiovascular risk factors may affect sperm quality, but there is limited scientific evidence in men.

**STUDY DESIGN, SIZE, DURATION:**

A cross-sectional analysis in the context of the Led-Fertyl (Lifestyle and Environmental Determinants of Seminogram and Other Male Fertility-Related Parameters) study was conducted.

**PARTICIPANTS/MATERIALS, SETTING, METHODS:**

A total of 223 young men aged 18–40 years were recruited between February 2021 and December 2024 in Reus (Catalonia, Spain). The AHA-LE8 ideal cardiovascular health (CVH) score (ranging from 0 to 100) was calculated as the means of eight CVH metrics and was considered as exposure. This score is based on four health behaviors (healthy diet, adequate physical activity, avoidance of nicotine, and healthy sleep) and four health factors (healthy weight, and healthy levels of blood lipids, blood glucose, and blood pressure). Conventional sperm quality parameters (count, concentration, vitality, total and progressive motility, and normal sperm morphology) were considered the main outcomes. Adherence to the LE8 score was categorized into tertiles using the lowest tertile as the reference (T1). All regression models were adjusted for several potential confounders: age (years), education (high school or less, college or higher education), monthly income (<2000 euros and ≥2000 euros), and sexual abstinence (days).

**MAIN RESULTS AND THE ROLE OF CHANCE:**

Compared with those in the lowest tertile, men in the highest tertile of the LE8 score had higher sperm concentration (*β* = 1.11; 95% CI: 0.12, 2.09), total motility (*β* = 6.05; 95% CI: 0.44, 11.65), and progressive motility (*β* = 5.84; 95% CI: 0.19, 11.48). In the continuous analysis, each 10-point increase in the LE8 score was positively associated with total sperm count (*β* = 0.88; 95% CI: 0.13, 1.63), sperm concentration (*β* = 0.45; 95% CI: 0.03, 0.86), total (*β* = 2.91; 95% CI: 0.56, 5.26), and progressive motility (*β* = 3.00; 95% CI: 0.63, 5.36). Compared with participants in the lowest tertile of the LE8 score, those in the highest tertile were 68% less likely to have an abnormal seminogram, according to the World Health Organization (2010) thresholds (OR: 0.32; 95% CI: 0.15, 0.67).

**LIMITATIONS, REASONS FOR CAUTION:**

Due to the cross-sectional design, a cause-effect relationship cannot be established. It is also not possible to generalize these results to other populations.

**WIDER IMPLICATIONS OF THE FINDINGS:**

Our findings suggest a positive association between the LE8 score and total sperm count, sperm concentration, total motility, and progressive motility. In addition, a higher LE8 score is associated with lower odds of an abnormal seminogram in healthy men of reproductive age.

**STUDY FUNDING/COMPETING INTEREST(S):**

The Led-Fertyl study was supported by the Spanish government’s official funding agency for biomedical research, Instituto de Salud Carlos III (ISCIII), through the Fondo de Investigacion para la Salud (FIS), the European Union ERDF/ESF, ‘A way to make Europe’/‘Investing in your future’ [PI21/01447], and the Diputació de Tarragona (2021/11-No.Exp. 8004330008-2021-0022642). J.S.-S. is a distinguished senior researcher supported by the ICREA Academia Program. E.D.-C. has received a Contrato Pre-doctoral de Formación en Investigación en Salud (PFIS FI22/00018) of the Acción Estratégica en Salud program (AES) from the ISCIII, Spanish Ministry of Health. All of the authors have no conflicts of interest to declare.

**TRIAL REGISTRATION NUMBER:**

N/A.

WHAT DOES THIS MEAN FOR PATIENTS? Infertility is a problem affecting 1 in 10 couples who want to have children. In nearly half of these cases, the issue lies with the man. Sperm quality can be affected by several factors. Some of these factors, such as genes, cannot be changed. However, others, such as lifestyle and daily habits, could play an important role. Unhealthy choices such as smoking, excessive alcohol consumption, not exercising enough, and eating poorly could reduce sperm quality. Health problems such as high blood pressure, diabetes, and high cholesterol may also have a negative effect.

In our study of 223 healthy men of reproductive age, we found that those who had a healthier lifestyle, including following a healthy diet, staying physically active, not smoking, sleeping well, maintaining a healthy body weight and controlling blood pressure, blood glucose, and cholesterol, tended to have better sperm quality. These habits are important not only for protecting the heart and preventing disease, but also for supporting male fertility.

## Introduction

Infertility is a disease of the reproductive system that affects both men and women (World Health Organization (WHO), 2018) and constitutes a global public health challenge. It is estimated to impact between 8% and 12% of couples of reproductive age, that represents over 48 million couples worldwide (The Lancet Global Health, 2022; World Health Organization (WHO), 2023), with male factors alone accounting to ∼20–30% of cases (Vander Borght and Wyns, 2018). Sperm quality analysis is one of the primarily methods used to assess male fertility (Schlegel *et al.*, 2021), and evidence suggests that sperm quality has steadily declined in recent years (Levine *et al.*, 2017, 2023).

Several factors have been recognized as contributors to the decline of male fertility and sperm quality, including environmental pollution (Pizzol *et al.*, 2021), psychological stress (Ilacqua *et al.*, 2018), and lifestyle factors such as unhealthy diet (Salas-Huetos *et al.*, 2017), sedentary behavior (Gaskins *et al.*, 2014), and smoking (Sharma *et al.*, 2016). Interestingly, the same factors are also associated with chronic diseases such as hypertension, type 2 diabetes mellitus (T2DM), hyperlipidemia, and obesity (Eisenberg *et al.*, 2016).

In 2022, the American Heart Association (AHA) developed the Life’s Essential 8 (LE8) score (Lloyd-Jones *et al.*, 2022), which incorporates sleep health and updates the previous Life’s Simple 7 score (LS7) (Lloyd-Jones *et al.*, 2010). LE8 is a composite score that integrates two domains: health behaviors (diet, physical activity, nicotine exposure, and sleep health) and health factors (BMI, blood lipids (non-high-density lipoprotein (HDL) cholesterol), blood glucose, and blood pressure). Recently, the LE8 score has been shown to be inversely associated with history of female infertility (Nichols *et al.*, 2024). However, to date, no studies have evaluated this association specifically in men. Until now, most studies on male fertility have looked at these factors individually. For example, meta-analyses of observational studies have shown that lower adherence to the Mediterranean diet (Cao *et al.*, 2022), high BMI (Li *et al.*, 2024), cigarette smoking (Bundhun *et al.*, 2019), excessive or high-intensity elite physical activity (>80 METs-h/week) (Ibañez-Perez *et al.*, 2019), and T2DM (Pergialiotis *et al.*, 2016), are all associated with lower sperm quality. However, studying these factors in combination through a lifestyle score like the LE8 may provide a more comprehensive perspective. In this sense, the present study aimed to investigate whether the LE8 score is associated or not with sperm quality parameters in healthy Spanish men of reproductive age from the Led-Fertyl cohort.

## Materials and methods

### Design and study population

The Led-Fertyl (Lifestyle and environmental determinants of seminogram and other male fertility-related parameters) study is a cross-sectional study designed to identify dietary and other lifestyle factors associated with sperm quality. A total of 226 participants were enrolled in the Led-Fertyl study between February 2021 and December 2024. Three participants were subsequently excluded due to azoospermia, resulting in a final sample size of 223 participants (Supplementary Fig. S1). The main inclusion criteria were that volunteers were healthy males aged 18–40 years from the general population. The exclusion criteria included: inability to attend scheduled intervention visits, institutionalization, severe chronic illness, a history of reproductive disease or vasectomy, a history of major organ transplantation, a documented history of cardiovascular disease, HIV positivity or hepatitis B or C, acute infection or inflammation, active cancer or a history of cancer within the last 5 years, severe psychiatric disorders, cirrhosis or liver failure. Participants were also excluded if they suffered from endocrine diseases or took antidepressants, calcium channel blockers, alpha-adrenergic blockers, antiepileptic drugs, antiretrovirals, immunosuppressive drugs, cytotoxic agents, systemic corticosteroids or if they had lost more than 5 kg in the last month. Participants were also excluded if they had a history of alcohol or drug abuse or had any condition that might interfere with their ability to comply with the study protocol. Once enrolled in the study, participants were asked to complete online self-report questionnaires and attend a face-to-face visit at the Hospital Universitari Sant Joan de Reus (Reus, Tarragona, Spain), where biological samples (blood and semen) were collected. All participants gave prior online and written informed consent to participate. This study was carried out according to the guidelines of the Declaration of Helsinki and the protocol was approved by the Ethics Committee of the Institut d’Investigació Sanitària Pere i Virgili (CEIm-IISPV, Ref. 181/2019).

### Exposure: LE8 score

The AHA LE8 score (Lloyd-Jones *et al.*, 2022) was calculated taking into account the eight most important predictors of heart health in two domains. First, the health behavior domain includes adherence to the Mediterranean diet considering the Mediterranean population included, physical activity, nicotine exposure, and sleep health. Second, the health factors domain includes BMI, blood lipids (non-HDL cholesterol), blood glucose, and blood pressure.

Blood pressure and body composition (weight, height, and waist circumference) were measured using standardized methods by trained personnel during a face-to-face visit. For blood pressure measurements, participants were seated comfortably in a quiet environment for 5 min before the procedure and blood pressure was measured in duplicate on the non-dominant arm, with a 5-min interval between measurements, using a semi-automatic oscillometer (Omron HEMs705CP, Hoofddorp, Netherlands). Weight was measured using a precision scale and an octopolar multifrequency bioelectrical impedance device (MC780MAS; Tanita, Tokyo, Japan). Height was measured using a portable stadiometer (SECA 213, Hamburg, Germany) according to standard procedures. BMI was calculated as weight (kg)/height (m^2^). Waist circumference was determined using a flexible, non-extensible measuring tape (Cescorf, Brasil). According to the Physical Activity Guidelines, the goal for physical activity was considered to be the equivalent of 150 min/week (U.S. Department of Health and Human Services, 2018), and was assessed using the validated Regicor Short Physical Activity Questionnaire (Molina *et al.*, 2017). Laboratory analyses were performed to determine plasma glucose concentrations and fasting total cholesterol levels, according to standard and validated protocols of the Hospital Universitari Sant Joan de Reus (Reus, Tarragona, Spain). Adherence to the Mediterranean diet was measured by trained dietitians’ interviewers using the validated Mediterranean Diet Adherence Screener (MEDAS) for the Spanish population (Schröder *et al.*, 2011). The sleep health metric was evaluated based on self-reported sleep duration. Socio-demographic, personal history, and lifestyle information were collected through online, self-administered questionnaires, which were subsequently reviewed for accuracy by trained staff. The scoring range for each of the eight cardiovascular health (CVH) components was from 0 to 100, following the methodology described by Lloyd-Jones *et al.* (2022). The overall LE8 score was calculated as the mean of these eight components, resulting in a total score of 0–100 for each participant, with higher scores indicating lower cardiovascular risk. The AHA defines three CVH categories based on the LE8 score: low (below 50), moderate (50–79), and high (80 and above). However, as no participant in our study population had LE8 scores below 50, we categorized LE8 adherence into tertiles based on the distribution of scores within our study sample. This ensures a balanced distribution of participants into the three categories and preserves statistical power in group comparisons. This approach enabled us to capture relative differences in adherence across the cohort. The lowest tertile (least healthy) was used as the reference category in the analyses.

### Outcome: sperm quality parameters

The methods used to analyze semen parameters (volume, pH, sperm count and concentration, sperm vitality, total and progressive sperm motility, and sperm morphology) have previously been described (Davila‐Cordova *et al.*, 2025) according to the WHO 5th edition of the laboratory manual for the examination and processing of human semen (World Health Organization, 2010).

Only complete semen samples—one per participant—were collected after a period of sexual abstinence of at least 3 days. All analyses were performed on fresh samples, within 60 min of collection. Semen volume and pH were measured after 20 min of liquefaction using pH indicator strips (Fisherbrand™, Madrid, Spain). The Computer-Aided Sperm Analysis (CASA) SCA^®^ (version 6.5.0.67, Microptic, Barcelona, Spain) in conjunction with an Olympus CX43 phase contrast microscope (EVIDENT Corporation, Tokyo, Japan) was used to assess sperm concentration, motility, vitality, and morphology. Total sperm count (millions of spermatozoa (spz) per ejaculate) was calculated by multiplying the ejaculate volume by the sperm concentration (millions of spermatozoa per milliliter (ml)). Sperm motility (expressed as a percentage) was measured using a 10× phase contrast objective and classified into progressive, non-progressive, and immotile spermatozoa. Total motility was expressed as the sum of progressive motility and non-progressive motility. Sperm vitality (expressed as %) was estimated using the hypo-osmotic swelling test and measured using a 60× objective. Sperm morphology (expressed as % of normal forms) was assessed on a smear of semen on a microscope slide, after air-drying, fixing, and staining with the Hemacolor^®^ kit (Sigma-Aldrich, Darmstadt, Germany). Morphology was evaluated with the 60× objective, identifying the percentage of normal and abnormal forms. Sperm motility, vitality, and morphology were analyzed in 200 spermatozoa.

Abnormal semen parameters were defined according to the WHO standards (World Health Organization, 2010), and included semen volume <1.5 ml, pH ≥7.2, sperm concentration <15 × 10^6^ spz/ml, sperm count <39 × 10^6^ spz, sperm vitality <58%, total and progressive sperm motility <40% and <32%, respectively, sperm morphology <4% normal forms, and an abnormal seminogram was considered if one or more these sperm parameters were outside the above reference limits.

Double-stranded DNA fragmentation in spermatozoa was assessed using the Comet assay under neutral conditions, as described by Ribas-Maynou *et al.* (2020). Agarose gel electrophoresis and SYTOX Orange staining were performed and analyzed using epifluorescence microscopy on at least 100 cells per sample. The degree of fragmentation was quantified using the Olive Tail Moment (OTM) parameter with Comet Score v2.0 software.

### Statistical analysis

Data analysis was conducted using the most recent version of the Led-Fertyl database, updated in December 2024. The sample size was calculated based on the assumption of 43.5% progressive sperm motility, 7% margin of error, and a 95% confidence level to account for subpopulations greater than 100 000 men and a minimum sample size was 192 participants. For the variable smoking status, 5.4% of the values were missing. These missing values were imputed by assigning the most frequent category (non-smokers).

Baseline characteristics of the study population were compared between LE8 score tertiles using one-way ANOVA or Kruskal–Wallis and chi-squared tests, as appropriate. Values are reported as percentages and numbers for categorical variables; and as means ± SDs or medians [25th, 75th percentiles] for continuous variables, depending on the normality of the distribution. Normality of variable distribution was assessed using the Kolmogorov–Smirnov test, and non-normal outcome variables were cubic root transformed to approach a Gaussian distribution.

Multivariable linear regression models were fitted to assess the associations between tertiles of the LE8 score and sperm quality parameters, including sperm DNA fragmentation (SDF), and were reported as *β*-coefficients and their 95% CIs. In addition, the LE8 score was also modeled as a continuous variable, so that the changes in sperm quality parameters and SDF for each 10-point increase in the LE8 score could be evaluated. These were also reported as *β*-coefficients and 95% CI. In the above linear regression analyses, square root transformed (total sperm count and concentration, vitality, and normal sperm morphology) and logarithmically transformed (SDF) were used. In addition, multivariable logistic regression models were fitted to evaluate the associations between the LE8 scores and abnormal sperm quality parameters according to WHO (2010) normality thresholds using the main outcomes of the total sperm count, sperm concentration, vitality, total and progressive motility and morphology, and expressed as odds ratios (OR) and 95% CIs (World Health Organization, 2010). All regression models were adjusted for several potential confounders: Model 1 was adjusted for age (years) and education (high school or less, college or higher education) and monthly income (<2000 euros or ≥2000 euros); and Model 2 was additionally adjusted for sexual abstinence (days).

An additional analysis was performed to assess the importance of each individual item of the LE8 score in the sperm quality parameters. Following a previously described method (Trichopoulou *et al.*, 2009), each item was removed one by one from the total score. By removing each individual item, the total score was reduced to seven items. Therefore, we multiplied the estimated *β*-coefficients by 7/8 to ensure comparability with the original score, which could have a maximum punctuation of 100.

Statistical analyses were performed using IBM-SPSS (version 27.0, SPSS Inc, Chicago, IL, USA) and Stata (version 14.0, StataCorp LLC, College Station, TX, USA). All tests were considered to be significant at a two-tailed *P*-value of <0.05.

## Results

### Descriptive results

The general characteristics of the study population are reported in Supplementary Table S1. The analysis included 223 male participants with a mean (±SD) age of 28.2 (±5.5) years. Most of them were single (87.0%) and had college or higher education (63.2%). Of the total number of participants, 10.8% fulfilled more than 11 points on the MEDAS, 92.8% performed the recommended amount of exercise (≥150 min/week), 74.9% were non-smokers, 75.8% had an ideal sleep health (7–<9 hours/day), 57.0% had a BMI <25 kg/m^2^, 70.4% had a non-HDL cholesterol level <130 mg/dl, 93.7% had a blood glucose concentration <100 mg/dl, and 22.9% had blood pressure <120/80 mmHg. In addition, 39.5% had at least one major seminogram parameter below the WHO (2010) reference values.

General characteristics according to tertiles of the LE8 score are reported in Table 1. Participants in the highest category (LE8 ≥ 88 points) were more likely to be younger and to have lower BMI, waist circumference, and seminogram abnormality, and higher sleep duration, and vitality, compared with the lowest category (LE8 < 79 points). In most of the LE8 score components, the percentage of values in the highest LE8 score tertile was progressively higher in the components of adherence to the Mediterranean diet, ideal physical activity, never smoking, ideal sleep health, ideal BMI, ideal non-HDL-cholesterol and blood pressure levels.

**Table 1.** General characteristics of the study population according to tertiles of Life’s Essential 8 score.

	Life’s Essential 8 score (0–100 points)			
General characteristics	T1	T2	T3	*P*-value
	<79	≥79 and <88	≥88	<0.001
	n = 76	n = 79	n = 68	
Age (years)	**29.3 ± 5.2**	**27.1 ± 5.4**	**28.3 ± 5.8**	**0.045**
BMI (kg/m^2^)	**26.5 ± 3.5**	**24.2 ± 2.9**	**22.9 ± 2.2**	**<0.001**
Waist circumference (cm)	**88.2 ± 8.9**	**82.6 ± 7.5**	**79.4 ± 5.9**	**<0.001**
Physical activity (METs-min/week)	3285 ± 2479	3776 ± 3440	4254 ± 2762	0.144
Sleep duration (hours/day)	**7.2 ± 0.9**	**7.4 ± 0.7**	**7.6 ± 0.5**	**0.003**
Education				
*High school or less*	30 (39.5)	31 (39.2)	21 (30.9)	0.482
*College or high education*	46 (60.5)	48 (60.8)	47 (69.1)	
Civil status				
*Single*	64 (84.2)	72 (91.1)	58 (85.3)	0.456
*Married*	11 (14.5)	7 (8.9)	8 (11.8)	
*Other*	1 (1.3)	0 (0)	2 (2.9)	
Monthly income				
*Less than 1000 euros*	8 (10.5)	11 (13.9)	13 (19.2)	0.133
*Between 1000 euros and 2000 euros*	55 (72.4)	52 (65.8)	35 (51.5)	
*More than 2000 euros*	13 (17.1)	16 (20.3)	20 (29.4)	
Life’s Essential 8 score				
*Ideal MEDAS ≥11 points*	**2 (2.6)**	**4 (5.1)**	**18 (26.5)**	**<0.001**
*Physical activity ≥150 min/week*	**71 (93.4)**	**74 (93.7)**	**62 (91.2)**	**<0.001**
* Never smoked*	**33 (43.4)**	**73 (92.4)**	**61 (89.7)**	**<0.001**
*Ideal sleep health*	**45 (59.2)**	**61 (77.2)**	**63 (92.7)**	**0.001**
* BMI <25 kg/m^2^*	**19 (25.0)**	**48 (60.8)**	**60 (88.2)**	**<0.001**
*Non-HDL cholesterol <130 mg/dl*	**38 (50.0)**	**58 (73.4)**	**61 (89.7)**	**<0.001**
*Blood glucose <100 mg/dl*	68 (89.5)	74 (93.7)	67 (98.5)	0.082
*Blood pressure <120/<80 mmHg*	**9 (11.8)**	**10 (12.7)**	**32 (47.1)**	**<0.001**
Seminogram parameters				
*Sexual abstinence (days)*	3.0 [3–4.8]	4 [3–5]	4 [3–5]	0.066
*pH*	8.5 [8.5–8.5]	8.5 [8.5–8.5]	8.5 [8–8.5]	0.137
*Volume (ml)*	3.4 [2.4–4.0]	3.5 [2.5–5.0]	3.6 [2.8–4.8]	0.327
*Volume <1.5 ml*	3 (4.0)	1 (1.3)	2 (2.9)	0.581
*Total sperm count (×10^6^ spz)*	130.8 [79.2–227.4]	184.3 [96.8–281.3]	175.5 [108.8–318.6]	0.060
*Total sperm count <39 × 10^6^ spz*	7 (9.2)	7 (8.9)	3 (4.4)	0.487
*Sperm concentration (×10^6^ spz/ml)*	42.7 [26.8–69.2]	42.5 [28.7–81.8]	62 [32.3–96.4]	0.059
*Sperm concentration <15 × 10^6^ spz/ml*	8 (10.5)	8 (10.1)	4 (5.9)	0.563
*Vitality (%)*	**80 [72.5–87.5]**	**83.5 [77.5–91.5]**	**85 [77.5–90]**	**0.035**
*Vitality <58%*	3 (4.0)	3 (3.8)	3 (4.4)	0.982
*Total motility (%)*	56.8 ± 16.9	60.7 ± 18.0	62.3 ± 14.3	0.112
*Total motility <40% motile*	11 (14.5)	10 (12.7)	5 (7.4)	0.390
*Progressive motility (%)*	40.2 ± 15.6	44.7 ± 17.6	45.0 ± 16.7	0.143
*Progressive motility <32% motile*	24 (31.6)	22 (27.9)	15 (22.1)	0.438
*Non-progressive motility (%)*	16.8 ± 5.4	16.6 ± 7.1	17.5 ± 7.3	0.714
*Normal sperm morphology (%)*	9 [5–15]	7.5 [4.5–14.5]	10 [5.3–14.8]	0.625
*Normal sperm morphology <4% normal*	12 (16.0)	8 (10.1)	7 (10.3)	0.458
*Seminogram abnormality*	**39 (51.3)**	**29 (36.7)**	**20 (29.4)**	**0.022**

HDL, high-density lipoprotein; MEDAS, Mediterranean diet adherence screener; METs, metabolic equivalents; n, number of subjects; spz, spermatozoa; T, tertiles. Continuous variables were presented as means (±SD) or medians [25th–75th percentiles] and categorical variables are presented as number (n) and percentages (%). One-way analysis of variance (ANOVA) or the Kruskal–Wallis test was used to evaluate differences across tertiles of Life’s Essential 8 score for normally and non-normally distributed continuous variables, respectively. Chi-squared test was used for comparisons between categorical variables. Bold indicates *P*-value <0.05.

### LE8 score and sperm quality parameters

Table 2 shows the associations (*β* coefficient; 95% CIs) between the LE8 score and sperm quality parameters. The fully adjusted multivariable linear regression model showed significantly higher total sperm count (*β* = 1.78; 95% CI: −0.02, 3.57, *P*-trend = 0.048), sperm concentration (*β* = 1.11; 95% CI: 0.12, 2.09, *P*-trend = 0.031), total motility (*β* = 6.05; 95% CI: 0.44, 11.65, *P*-trend = 0.033), and progressive motility (*β* = 5.84; 95% CI: 0.19, 11.48, *P*-trend = 0.037) in participants in the highest LE8 score tertile, compared to those participants in the lowest tertile. In the continuous analysis, each 10-point increase in the LE8 score was positively associated with total sperm count (*β* = 0.88; 95% CI: 0.13, 1.63), sperm concentration (*β* = 0.45; 95% CI: 0.03, 0.86), total (*β* = 2.91; 95% CI: 0.56, 5.26), and progressive motility (*β* = 3.00; 95% CI: 0.63, 5.36) (Table 2). When SDF was examined as an outcome, regression analyses indicated no significant associations between adherence to the LE8 score and SDF (*P* > 0.05) (Supplementary Table S2).

**Table 2.** Association (*β* coefficients and their 95% confidence interval) across tertiles and in continuous for each 10-point increase in the Life’s Essential 8 score and sperm quality parameters.

Sperm quality parameters		Life’s Essential 8 score				
		T1	T2	T3	*P*-trend	Continuous
Total sperm count (×10^6^ spz)^1^	Crude model	Ref	0.98 (−0.75,2.70)	**2.02 (0.23,3.80)**	**0.027**	**0.82 (0.07,1.56)**
	Model 1	Ref	1.37 (−0.38,3.12)	**2.29 (0.48,4.10)**	**0.013**	**1.04 (0.27,1.80)**
	Model 2	Ref	1.18 (−0.53,2.89)	1.78 (−0.02,3.57)	**0.048**	**0.88 (0.13,1.63)**
Sperm concentration (×10^6^ spz/ml)^1^	Crude model	Ref	0.30 (−0.63,1.22)	**1.20 (0.24,2.16)**	**0.017**	**0.43 (0.03,0.83)**
	Model 1	Ref	0.42 (−0.52,1.36)	**1.26 (0.28,2.24)**	**0.013**	**0.50 (0.08,0.91)**
	Model 2	Ref	0.36 (−0.58,1.30)	**1.11 (0.12,2.09)**	**0.031**	**0.45 (0.03,0.86)**
Sperm vitality (%)^1^	Crude model	Ref	0.19 (−0.03,0.41)	0.21 (−0.02,0.44)	0.059	**0.10 (0.01,0.19)**
	Model 1	Ref	0.16 (−0.06,0.39)	0.20 (−0.04,0.43)	0.089	0.08 (−0.02,0.18)
	Model 2	Ref	0.16 (−0.06,0.39)	0.20 (−0.04,0.44)	0.087	0.08 (−0.02,0.18)
Total motility (%)	Crude model	Ref	3.98 (−1.25,9.22)	**5.58 (0.14,11.02)**	**0.040**	**2.85 (0.59,5.11)**
	Model 1	Ref	3.65 (−1.68,8.97)	**5.91 (0.39,11.44)**	**0.034**	**2.88 (0.55,5.21)**
	Model 2	Ref	3.70 (−1.65,9.04)	**6.05 (0.44,11.65)**	**0.033**	**2.91 (0.56,5.26)**
Progressive motility (%)	Crude model	Ref	4.52 (−0.76,9.79)	4.81 (−0.67,10.28)	0.071	**2.64 (0.36,4.91)**
	Model 1	Ref	4.69 (−0.67,10.05)	**5.58 (0.02,11.14)**	**0.043**	**2.92 (0.58,5.26)**
	Model 2	Ref	4.79 (−0.59,10.17)	**5.84 (0.19,11.48)**	**0.037**	**3.00 (0.63,5.36)**
Non-progressive motility (%)	Crude model	Ref	−0.18 (−2.28,1.92)	0.68 (−1.50,2.86)	0.572	0.20 (−0.71,1.11)
	Model 1	Ref	−0.73 (−2.86,1.40)	0.27 (−1.94,2.47)	0.865	−0.07 (−1.00,0.87)
	Model 2	Ref	−0.74 (−2.88,1.39)	0.24 (−2.01,2.48)	0.894	−0.08 (−1.02,0.86)
Normal sperm morphology (%)^1^	Crude model	Ref	−0.09 (−0.44,0.26)	0.08 (−0.29,0.44)	0.740	0.06 (−0.09,0.22)
	Model 1	Ref	−0.10 (−0.46,0.27)	0.07 (−0.30,0.45)	0.743	0.07 (−0.09,0.22)
	Model 2	Ref	−0.08 (−0.44,0.28)	0.12 (−0.26,0.50)	0.588	0.08 (−0.08,0.24)

spz, spermatozoa, T, Tertile. β coefficients were estimated using multivariable linear regression models. Model 1 was adjusted for age (years), education (high school or less, college or high education), and monthly income (<2000 euros, ≥2000 euros). Model 2 was additionally adjusted for sexual abstinence (days). ^1^Total sperm count, sperm concentration, sperm vitality, and normal sperm morphology were root-squared transformed data to approximate a normal distribution. Bold indicates *P*-value <0.05.

Using a multivariable logistic regression model, compared with participants in the lowest tertile of the LE8 score adherence, those in the highest tertile were 68% less likely to have an abnormal seminogram according to the WHO (2010) guidelines (OR: 0.32; 95% CI: 0.15, 0.67) (Supplementary Fig. S2).

Table 3 reports the associations between the LE8 score—modeled as a continuous variable- and sperm quality parameters, when each individual component of the 8-item LE8 score was removed one by one. Compared to the overall LE8 score, we observed that excluding the MEDAS component substantially attenuated the positive associations found with total sperm count, sperm concentration, and total motility. Specifically, these associations were reduced by 38.6% (*β* = 0.54; 95% CI: −0.17, 1.25), 31.1% (*β* = 0.31; 95% CI: −0.09, 0.70), and 24.7% (*β* = 2.19; 95% CI: −0.03, 4.40), respectively. Similarly, removing the blood pressure component attenuated the positive associations with sperm concentration, total motility, and progressive motility by 31.1% (*β* = 0.31; 95% CI: −0.04, 0.67), 43.3% (*β* = 1.65; 95% CI: −0.36, 0.67), and 35.0% (*β* = 1.95; 95% CI: −0.08, 3.97), respectively. When the blood lipids component was removed, the positive associations with total sperm count and sperm concentration were also attenuated, by 37.5% (*β* = 0.55; 95% CI: −0.07, 1.17) and 26.7% (*β* = 0.33; 95% CI: −0.01, 0.67), respectively. Finally, when the BMI component was removed, the positive association with sperm concentration was attenuated by 20.0% (*β* = 0.36; 95% CI: −0.01, 0.72). No substantial changes were observed when the physical activity, smoking, sleep health, or blood glucose components were excluded.

**Table 3.** Association between each 10-point increase in the 8-item Life’s Essential 8 score and changes in sperm quality parameters after alternate subtraction of each of its Life’s Essential 8 components.

	8-item Life’s Essential 8	Minus item 1 (MEDAS)	Minus item 2 (physical activity)	Minus item 3 (smoking)	Minus item 4 (sleep health)	Minus item 5 (BMI)	Minus item 6 (blood lipids)	Minus item 7 (blood glucose)	Minus item 8 (blood pressure)
Total sperm count (×10^6^ spz)	**0.88 (0.13, 1.63)**	0.54 (−0.17, 1.25)	**0.66 (0.09, 1.23)**	**1.00 (0.35, 1.67)**	**0.78 (0.18, 1.39)**	**0.76 (0.11, 1.41)**	0.55 (−0.07, 1.17)	**0.62 (0.03, 1.20)**	**0.71 (0.07, 1.35)**
Reduction/increase (%)		−38.6	**−25.0**	**+13.6**	**−11.4**	**−13.6**	−37.5	**−29.6**	**−19.3**
Sperm concentration (×10^6^ spz/ml)	**0.45 (0.03, 0.86)**	0.31 (−0.09, 0.70)	**0.33 (0.01, 0.65)**	**0.46 (0.10, 0.82)**	**0.42 (0.09, 0.75)**	0.36 (−0.01, 0.72)	0.33 (−0.01, 0.67)	**0.33 (0.01, 0.65)**	0.31 (−0.04, 0.67)
Reduction/increase (%)		−31.1	**−26.7**	**+2.2**	**−6.7**	−20.0	−26.7	**−26.7**	−31.1
Sperm vitality (%)	0.08 (−0.02, 0.18)	0.08 (−0.01, 0.17)	0.07 (−0.01, 0.14)	0.06 (−0.03, 0.14)	0.08 (−0.00, 0.16)	0.08 (−0.01, 0.16)	0.05 (−0.03, 0.13)	0.07 (−0.01, 0.14)	0.06 (−0.03, 0.14)
Reduction/increase (%)		0	−12.5	−25.0	0	0	−37.5	−12.5	−25.0
Total motility (%)	**2.91 (0.56, 5.26)**	2.19 (−0.03, 4.40)	**2.21 (0.42, 4.00)**	**3.33 (1.30, 5.37)**	**2.51 (0.62, 4.40)**	**2.43 (0.38, 4.48)**	**2.07 (0.13, 4.01)**	**2.17 (0.35, 3.99)**	1.65 (−0.36, 0.67)
Reduction/increase (%)		−24.7	**−24.0**	**+14.4**	**−13.7**	**−16.5**	**−28.9**	**−25.4**	−43.3
Progressive motility (%)	**3.00 (0.63, 5.36)**	**2.63 (0.41, 4.85)**	**2.21 (0.41, 4.01)**	**3.34 (1.29, 5.39)**	**2.47 (0.56, 4.37)**	**2.29 (0.22, 4.35)**	**2.06 (0.11, 4.02)**	**2.25 (0.42, 4.08)**	1.95 (−0.08, 3.97)
Reduction/increase (%)		**−12.3**	**−26.3**	**+11.3**	**−17.7**	**−23.7**	**−31.3**	**−25.0**	−35.0
Non-progressive motility (%)	−0.08 (−1.02, 0.86)	−0.24 (−1.13, 0.64)	−0.01 (−0.73, 0.71)	−0.01 (−0.81, 0.84)	0.00 (−0.76, 0.76)	0.11 (−0.71, 0.93)	−0.10 (−0.88, 0.67)	−0.08 (−0.81, 0.65)	−0.24 (−1.04, 0.56)
Reduction/increase (%)		NA	−87.5	−87.5	NA	NA	+25.0	0	NA
Normal sperm morphology (%)	0.08 (−0.08, 0.24)	0.03 (−0.12, 0.18)	0.06 (−0.07, 0.18)	0.09 (−0.05, 0.23)	0.07 (−0.06, 0.20)	0.07 (−0.07, 0.20)	0.03 (−0.10, 0.16)	0.05 (−0.07, 0.17)	0.10 (−0.03, 0.24)
Reduction/increase (%)		−62.5	−25.0	+12.5	−12.5	−12.5	−62.5	−37.5	+25.0

MEDAS, Mediterranean diet adherence screener and spz, spermatozoa. *β* coefficients and 95% CI are presented and were estimated using continuous analysis for each 10-point increase. Models were adjusted for age (years), education (high school or less, college or high education), and monthly income (<2000 euros, ≥2000 euros), and sexual abstinence (days). For each sperm quality parameter, the Life’s Essential 8 score and it changes (reduction or increase) corresponding to subtracting the score components are shown.

## Discussion

To the best of our knowledge, the present study is the first to analyze the associations between the LE8 score, including lifestyle and cardiometabolic factors, and sperm quality parameters in healthy volunteers of reproductive age. Our main findings showed a positive association between the LE8 score and several sperm quality parameters including total sperm count, concentration, and motility. These associations were independent of potential confounders such as age, education, monthly income, and sexual abstinence. Secondly, we found that, compared to participants in the lowest tertile of the LE8 score, participants in the highest tertile had 68% lower odds of having an abnormal seminogram. Finally, after alternatively removing individual components of the LE8 score, we found that adherence to the Mediterranean diet, blood pressure, blood lipids, and BMI were the major contributors to the positive associations between the LE8 score and sperm quality parameters.

To date, no studies have specifically examined the relationship between the LE8 score and sperm quality. However, research in women has investigated the association of LS7 (Wang *et al.*, 2024), LE8 (Nichols *et al.*, 2024), and the Healthy Lifestyle Score (HLS) (Huang *et al.*, 2024) with fertility outcomes. Notably, lower LS7, LE8, and HLS scores have been linked to a higher likelihood of infertility in women. It is worth mentioning that the HLS score was determined using a 17-item questionnaire covering factors such as the lifetime number of sexual partners, the timing of menstruation onset and cessation, reproductive history, surgeries involving the genital tract, abnormal pregnancy events, and contraceptive use (Huang *et al.*, 2024). Although our study did not include information on fertility diagnoses, these findings in women highlight the importance of exploring similar associations between lifestyle scores and reproductive health in men.

Nevertheless, there is scientific evidence from observational and cross-sectional studies that relate each of the components of the LE8 score separately to sperm quality parameters. A meta-analysis of six cross-sectional studies involving 1244 subjects found that greater adherence to the Mediterranean diet was significantly associated with higher total count, sperm concentration, and progressive motility (Cao *et al.*, 2022). These findings align with results from a previous study by our group (Davila‐Cordova *et al.*, 2025). Regarding physical activity, a systematic review leading to a meta-analysis of 32 studies found contradictory results regarding the associations between different types of activity and sperm quality. While moderate physical activity (20–80 METs-h/week) appears to be positively associated with sperm quality, excessive or high-intensity, such as elite-level physical activity (>80 METs-h/week), is negatively associated (Ibañez-Perez *et al.*, 2019). In terms of smoking, Bundhun *et al.* (2019) conducted a meta-analysis of 16 studies including a total of 10 823 infertile men (5257 smokers and 5566 non-smokers) and reported that smoking was associated with a lower total sperm count and an increase in sperm morphology defects. Regarding overweight or obesity, a recent meta-analysis (Li *et al.*, 2024) including 50 studies with a total of 71 337 participants observed that men who were overweight or obese, based on BMI ≥25 kg/m^2^, have lower sperm quality. In addition to the aforementioned meta-analyses, total cholesterol levels were positively associated with normal sperm morphology (Schisterman *et al.*, 2014; Zhang *et al.*, 2017). Moreover, a meta-analysis of 12 observational studies including 1151 individuals with T2DM and 664 controls, concluded that sperm motility is lower in men with T2DM compared to controls (Pergialiotis *et al.*, 2016). Finally, a negative association between poor (<6 h/day) and long (>9 h/day) sleep duration and sperm quality parameters (Chen *et al.*, 2020), and high blood pressure and low total sperm count and total motility has been reported (Guo *et al.*, 2017). Taken together, this evidence is consistent with the findings of the present study, in which the stepwise removal of individual components from the LE8 score—particularly Mediterranean diet adherence, blood pressure, blood lipids, and BMI—attenuated the association between the LE8 score and sperm quality parameters.

The positive association observed between higher adherence to the LE8 score and improved sperm quality parameters can be attributed to several interrelated biological mechanisms. In our study, this relationship was partly explained by the specific dietary patterns followed by the participants. Adherence to healthy diets such as the Mediterranean diet and the Healthful Plant-Based Diet Index, both rich in fruit, vegetables, whole grains, legumes, and healthy fats, has been previously linked to improved sperm quality parameters (Davila-Cordova *et al.*, 2025). Such diets are naturally high in antioxidants (Salas-Huetos *et al.*, 2017) and anti-inflammatory compounds (Fung *et al.*, 2005), which may counteract oxidative stress, a key cause of sperm DNA damage (Lanzafame *et al.*, 2009) and impaired spermatogenesis (Alahmar, 2019).

In addition to dietary factors, other LE8 components, such as regular physical activity, adequate sleep, and avoiding smoking, may also contribute to a favorable oxidative and hormonal environment for spermatogenesis (Sharma *et al.*, 2013; Aitken and Baker, 2020; Leisegang *et al.*, 2021). Moreover, optimal metabolic health, reflected by normal levels of BMI, glucose, lipids, and blood pressure, has also been linked to better testicular function, endocrine balance, and spermatogenic function (Kupelian *et al.*, 2008; Pakpahan *et al.*, 2023; Budihastuti *et al.*, 2024).

However, we observed no significant association between adherence to the LE8 score and SDF, a finding consistent with previous literature reporting inconsistent or null associations between lifestyle indices and SDF (Inversetti *et al.*, 2025). This null finding suggests that, while the LE8 score captures key health behaviors and factors relevant to general sperm function, it may not sufficiently reflect the specific mechanisms of sperm genomic integrity. There is growing evidence that SDF plays a critical role in male infertility and adverse reproductive outcomes, including idiopathic recurrent pregnancy loss (Busnelli *et al.*, 2023; Inversetti *et al.*, 2025). Factors not captured within the LE8 framework, such as environmental pollution (Bosco *et al.*, 2018), deficiencies in specific antioxidants and micronutrients (e.g. folate and zinc), and clinical urogenital conditions (e.g. varicocele and chronic inflammation) (Fomichova *et al.*, 2025), may play a more direct role in SDF.

Our study has some limitations that deserve to be mentioned. First, the cross-sectional design of our study precludes the ability to establish causal relationships between adherence to the LE8 score and sperm quality parameters. Secondly, although the study aimed to include participants from the general population, it was conducted in a relatively homogeneous sample of young, single, well-educated men residing in Spain, particularly in the Catalonia region. Furthermore, the Mediterranean diet is one of the LE8 dietary components, we recognize that this dietary pattern is region-specific and may not reflect the dietary habits of populations in other parts of the world. Consequently, these findings cannot be generalized to populations with different demographic or cultural characteristics. For this reason, we emphasize the need to replicate these results in other cohorts.

The main strength is that this is the first study to evaluate the association between the LE8 score and sperm quality parameters, filling an important gap in the literature. In addition, sperm quality analysis was performed using the CASA SCA^®^ system, which reduces subjectivity and variability in the analysis. Furthermore, we can highlight the adjustment of our analyses for several confounding variables that might cause potential bias. Finally, the LE8 instrument is a simple and cost-effective tool for the assessment of the quality of life and the cardiovascular risk of the population.

## Conclusions

In conclusion, our study suggests that higher LE8 scores are positively associated with total sperm count, sperm concentration, total and progressive motility. In addition, a higher LE8 score is associated with 68% lower odds of having an abnormal seminogram in healthy men of reproductive age. This study could provide valuable insights into the importance of a healthy lifestyle for both CVH and male reproductive health. However, further research from prospective and intervention studies are needed to confirm these results and explore causal relationships.

## Supplementary Material

hoaf059_Supplementary_Data

## Data Availability

The data underlying this article are available from the corresponding author upon request.
