# Supplementary material for: Life’s Essential 8 score and its association with sperm quality parameters in reproductive-aged men: evidence from the Led-Fertyl cohort
Source: Hum Reprod Open. 2025 Sep 18;2025(4):hoaf059. doi: 10.1093/hropen/hoaf059 (PMC12501419; doi:10.1093/hropen/hoaf059)
Supplement: hoaf059_Supplementary_Data [file hoaf059_supplementary_data.zip › HROPEN-25-0250.R2_Supplementary_Figure_S1-S2_EO.docx]

Assessed for eligibility (n=350)

Excluded (n=98):

- Declined to participate (n=86)
- To meet exclusion criteria (n=12)

Selected participants (n=252)

Dropped out (n=26)

Excluded participants (n=3)

- Participants with Azoospermia (n=3)

Included in analysis (n=223)

Supplementary Figure S1. Selection of participants for analysis


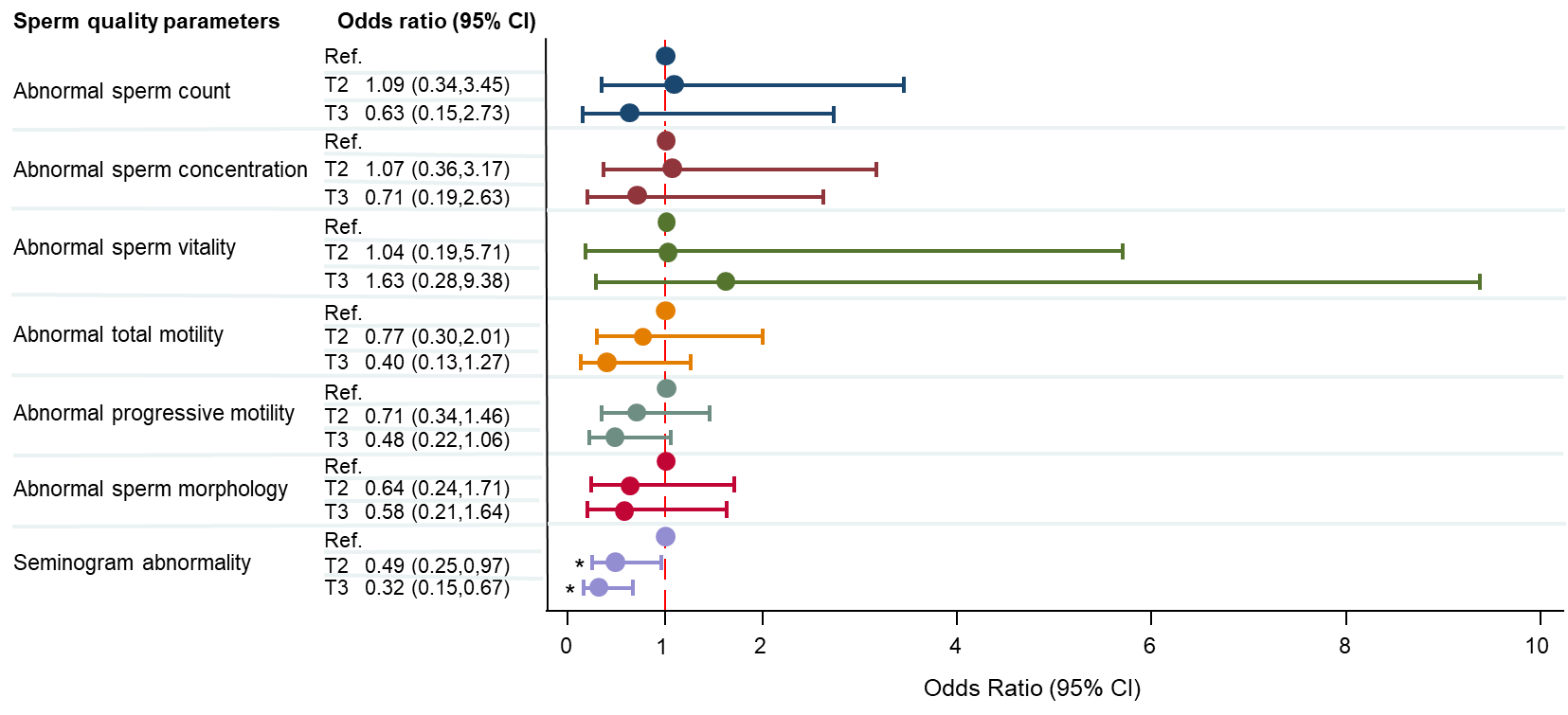


Supplementary Figure S2. Associations (OR and its 95% confidence interval) between Life’s Essential 8 score in tertiles and abnormal sperm quality parameters (n = 223). Odds ratios were estimated using multivariable logistic regression models. Models were adjusted for age (years), education (high school or less, college or high education), monthly income (<2000 euros, ≥2000 euros), and sexual abstinence (days). *Asterisks denote statistical significance in differences between T3 and T1 (reference).
