# Supplementary material for: Life’s Essential 8 score and its association with sperm quality parameters in reproductive-aged men: evidence from the Led-Fertyl cohort
Source: Hum Reprod Open. 2025 Sep 18;2025(4):hoaf059. doi: 10.1093/hropen/hoaf059 (PMC12501419; doi:10.1093/hropen/hoaf059)
Supplement: hoaf059_Supplementary_Data [file hoaf059_supplementary_data.zip › Supplementary Table S1, S2.docx]

| Supplementary Table S1. General characteristics of the study population | |
| --- | --- |
| General characteristics | **All population (n=223)** |
| Age (years) | 28.2 ± 5.5 |
| BMI (kg/m^2^) | 24.6 ± 3.3 |
| Waist circumference (cm) | 83.5 ± 8.3 |
| Systolic blood pressure (mmHg) | 127 ± 10 |
| Diastolic blood pressure (mmHg) | 74 ± 9 |
| Physical activity (METs-min/week) | 3754 ± 2948 |
| Sleep duration (hours/day) | 7.4 ± 0.8 |
| Education |  |
| High school or less | 82 (36.8) |
| College or high education | 141 (63.2) |
| Civil status |  |
| Single | 194 (87.0) |
| Married | 26 (11.7) |
| Other | 3 (1.4) |
| Monthly income |  |
| Less than 1000 € | 32 (14.4) |
| Between 1000 € and 2000 € | 142 (63.7) |
| More than 2000 € | 49 (22.0) |
| Life’s Simple 7 |  |
| Ideal MEDAS ≥ 11 points | 24 (10.8) |
| Physical activity ≥ 150 min/week | 207 (92.8) |
| Never smoker | 167 (74.9) |
| Ideal sleep health (7-<9 hours/day) | 169 (75.8) |
| BMI < 25 kg/m^2^ | 127 (57.0) |
| Non-HDL cholesterol < 130 mg/dL | 157 (70.4) |
| Blood glucose < 100 mg/dL | 209 (93.7) |
| Blood pressure < 120/<80 mmHg | 51(22.9) |
| Seminogram parameters |  |
| Sexual abstinence (days) | 4 [3-5] |
| pH | 8.5 [8.0-8.5] |
| Volume (ml) | 3.5 [2.5-4.5] |
| Volume <1.5 ml | 6 (2.7) |
| Total sperm count (x10^6^) | 160.7 [98.0-272.5] |
| Total sperm count <39 × 10^6^ spz | 17 (7.6) |
| Sperm concentration (x10^6^/ml) | 48.1 [29.0-81.8] |
| Sperm concentration <15 × 10^6^ spz/ml | 20 (9.0) |
| Vitality (%) | 83.0 [76.0-90.0] |
| Vitality <58% | 9 (4.1) |
| Total motility (%) | 59.9 ± 16.6 |
| Total motility <40% motile | 26 (11.7) |
| Progressive motility (%) | 43.3 ± 16.7 |
| Progressive motility <32% motile | 61 (27.4) |
| Non-progressive motility (%) | 16.9 ± 6.6 |
| Normal sperm morphology (%) | 8.5 [5.0-15.0] |
| Normal sperm morphology <4% normal | 27 (12.2) |
| Seminogram abnormality | 88 (39.5) |
| Abbreviations. BMI, Body mass index; MEDAS, Mediterranean diet adherence screener; METs, Metabolic equivalents; n, number of subjects; and spz, spermatozoa. Continuous variables were presented as means (±SD) or medians [25th-75th percentiles] and categorical variables are presented as number (n) and percentages (%). | |

| Supplementary Table S2. Association (β coefficients and their 95% confidence interval) across tertiles and in continuous for each 10-point increase in the Life’s Essential 8 score and sperm DNA fragmentation. | | | | | | |
| --- | --- | --- | --- | --- | --- | --- |
|  | | **Life’s Essential 8 score** | | | | |
|  |  | T1 | T2 | T3 | p-trend | Continuous |
| Sperm DNA fragmentation* | Crude model | Ref | 0.15 (-0.07,0.37) | 0.07 (-0.16,0.30) | 0.491 | 0.00 (-0.01,0.01) |
|  | Model 1 | Ref | 0.17 (-0.06,0.39) | 0.08 (-0.15,0.31) | 0.426 | 0.00 (-0.01,0.01) |
|  | Model 2 | Ref | 0.17 (-0.05,0.40) | 0.09 (-0.15,0.33) | 0.388 | 0.00 (-0.01,0.01) |
| Abbreviations. T, Tertile. β coefficients were estimated using multivariable linear regression models. Model 1 was adjusted for age (years), education (high school or less, college or high education), and monthly income (<2000 €, ≥2000 €). Model 2 was additionally adjusted for sexual abstinence (days). ^*^ Sperm DNA fragmentation data were logarithmically transformed to approximate a normal distribution. | | | | | | |
